# Defective Lamin A-Rb Signaling in Hutchinson-Gilford Progeria Syndrome and Reversal by Farnesyltransferase Inhibition

**DOI:** 10.1371/journal.pone.0011132

**Published:** 2010-06-15

**Authors:** Jackleen Marji, Seán I. O'Donoghue, Dayle McClintock, Venkata P. Satagopam, Reinhard Schneider, Desiree Ratner, Howard J. Worman, Leslie B. Gordon, Karima Djabali

**Affiliations:** 1 Department of Dermatology, College of Physicians and Surgeons, Columbia University, New York, New York, United States of America; 2 EMBL, Heidelberg, Germany; 3 Departments of Medicine and of Pathology and Cell Biology, College of Physicians and Surgeons, Columbia University, New York, New York, United States of America; 4 Department of Pediatrics, Warren Albert Medical School of Brown University, Providence, Rhode Island, United States of America; 5 Department of Dermatology, Technical University Munich, Munich, Germany; Roswell Park Cancer Institute, United States of America

## Abstract

Hutchinson-Gilford Progeria Syndrome (HGPS) is a rare premature aging disorder caused by a *de novo* heterozygous point mutation G608G (GGC>GGT) within exon 11 of *LMNA* gene encoding A-type nuclear lamins. This mutation elicits an internal deletion of 50 amino acids in the carboxyl-terminus of prelamin A. The truncated protein, progerin, retains a farnesylated cysteine at its carboxyl terminus, a modification involved in HGPS pathogenesis. Inhibition of protein farnesylation has been shown to improve abnormal nuclear morphology and phenotype in cellular and animal models of HGPS. We analyzed global gene expression changes in fibroblasts from human subjects with HGPS and found that a lamin A-Rb signaling network is a major defective regulatory axis. Treatment of fibroblasts with a protein farnesyltransferase inhibitor reversed the gene expression defects. Our study identifies Rb as a key factor in HGPS pathogenesis and suggests that its modulation could ameliorate premature aging and possibly complications of physiological aging.

## Introduction

Hutchinson–Gilford progeria syndrome (HGPS) is a rare, sporadic genetic disorder with phenotypic features of premature aging [1] [2], [3], [4]. It is caused by *de novo* dominant mutations in *LMNA* [5], [6], [7]. *LMNA* encodes A-type nuclear lamins, with the predominant somatic cell isoforms lamin A and lamin C arising by alternative RNA splicing [8]. Lamins are intermediate filament proteins that polymerize to form the nuclear lamina, a meshwork associated with the inner nuclear membrane. HGPS is one of a spectrum of diverse diseases, sometimes referred to as “laminopathies,” caused by mutations in *LMNA* [9].

Lamin A is synthesized as a precursor, prelamin A, which has a CaaX motif at its carboxyl terminus. The CaaX motif signals a series of catalytic reactions resulting in a carboxyl-terminal cysteine that is farnesylated and carboxymethylated [9]. Farnesylated, carboxymethylated prelamin A is normally cleaved near its carboxyl-terminus in a reaction catalyzed by ZMPSTE24 endoprotease, leading to removal of the farnesylated cysteine [9]. The *LMNA* G608G mutation responsible for the majority of cases of HGPS creates an abnormal splice donor site within exon 11, generating an mRNA that encodes a prelamin A with a 50 amino acid deletion at its carboxyl-terminal domain [5], [6]. The ZMPSTE24 endoproteolytic site is deleted from progerin and hence retains a farnesylated and carboxymethylated cysteine at its carboxyl terminus [9]. Expression of progerin induces severe abnormalities in nuclear morphology, heterochromatin organization, mitosis, DNA replication and DNA repair [5], [6], [10], [11], [12], [13], [14], [15]. Progerin toxicity is attributed at least in part to its farnesyl moiety, as chemical inhibitors of protein farnesyltransferase (FTIs) reverse abnormalities in nuclear morphology in progerin expressing cells [16], [17], [18], [19], [20]. In addition, FTIs and other chemical inhibitors of protein prenylation partially reverse progeria-like phenotypes in genetically modified mice that express progerin or lack ZMPSTE24, and therefore accumulate unprocessed, farnesylated prelamin A [21], [22], [23], [24].

While several studies have clearly implicated farnesylated progerin in HGPS, the precise molecular mechanisms of how it induces HGPS pathology remain to be understood. Initial gene expression profiling of fibroblasts from human subjects with progeria syndromes and transfected cell models identified changes in sets of genes implicated in diverse pathways that have not always been consistent and have not been shown to be reversed by interventions such as treatment with FTIs [25], [26], [27], [28]. Therefore, we carried out additional genome-wide expression studies in cells from children with HGPS to identify alterations in functional groups of genes that define defective signaling pathways and to determine if FTI treatment reverses these defects. Our results demonstrate a link between progerin and the retinoblastoma protein (Rb) signaling pathway in HGPS.

## Results

### Lamin A-Rb signaling network is implicated in HGPS pathophysiology

**Figure 1:**
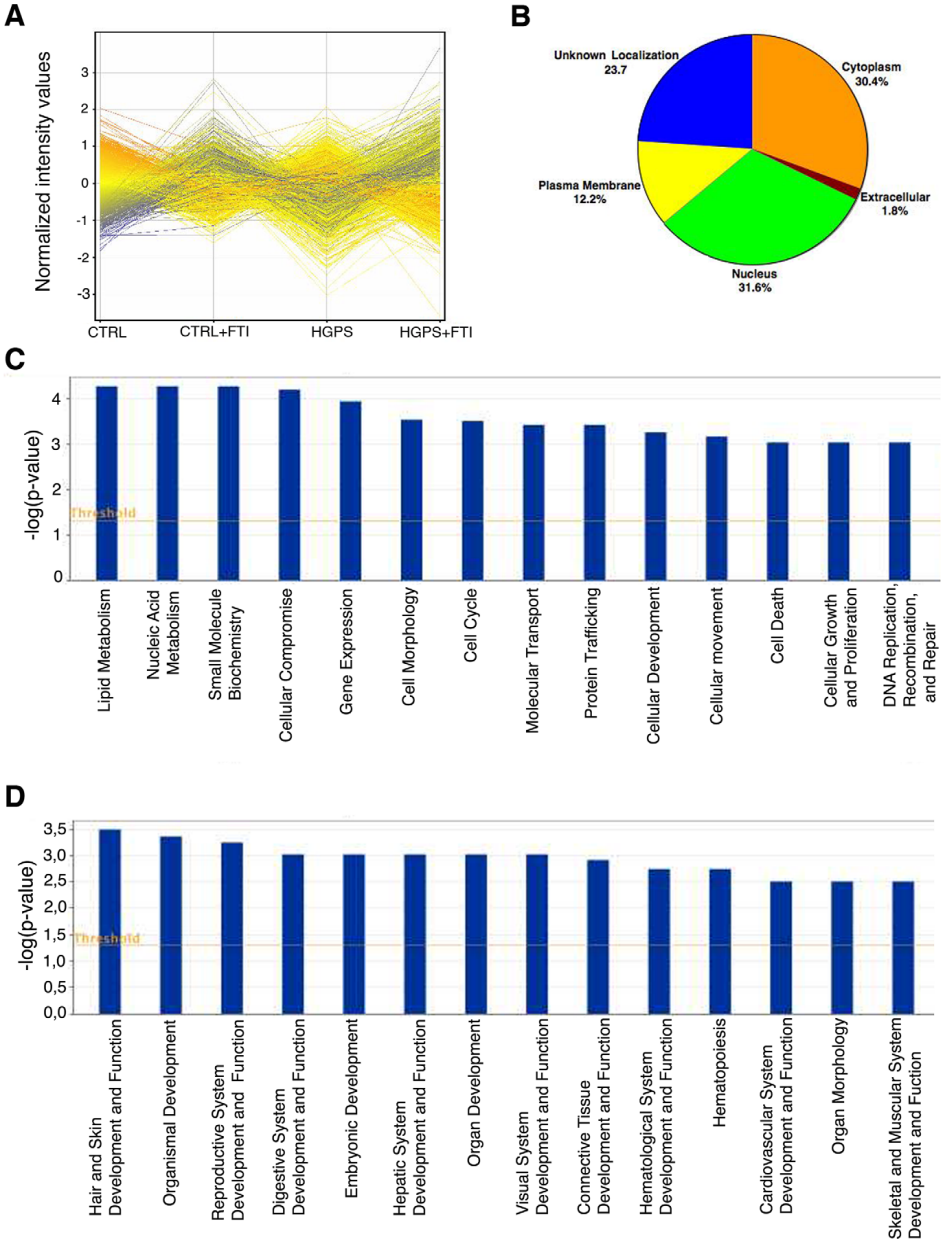
Genome-wide expression profiling of HGPS and control fibroblast cultures. (A) Microarray plot profiles indicate changes in gene expression in control, HGPS, FTI-treated control and FTI-treated HGPS fibroblasts. Each continuous line corresponds to the normalized intensity value of an individual probe set. Line colors denote the intensity of the signal (red: strong and blue: low signal). Probes that satisfied a greater or less than two-fold cutoff and statistically significant difference of p <0.01 are displayed. (B) Pie chart indicates the predicted subcellular localization of proteins encoded by the 352 genes differentially expressed in HGPS. The list of differentially expressed genes in HGPS versus control cells was analyzed using Ingenuity Pathway Analysis (IPA) and encoded proteins assigned a subcellular localization based on information contained in the Ingenuity Knowledge Base. (C) Genes differently expressed in HGPS (352 genes) were assigned to diverse cellular functions using the “Functional Analysis” tool of IPA software (www.ingenuity.com). Columns represent groups of genes associated with specific cellular functions (*x*-axis). The significant genes were compared to IPA database and ranked according to a p-values generated with Fisher extract test. P-values less than 0.05 indicates a statistically significant, non-random association between a set of significant genes and a set of all genes related to a given function in IPA database. The ratio (*y*-axis) represents the number of genes from the dataset that map to the pathway divided by the number of all known genes ascribed to the pathway. The yellow line represents the threshold of *p*<0.05. (D) As described above, the 352 genes were assigned to diverse physiological systems according to IPA.

To determine the mechanisms by which progerin exerts its pathological effect, we performed parallel microarray analyses of fibroblasts from subjects with HGPS and control individuals that were treated or untreated with the FTI lonafarnib for three days. We used RNA isolated from fibroblasts from five subjects with HGPS and five unaffected individuals to hybridize Affymetrix U133 plus 2.0 arrays. We identified 50,636 probe sets (Fig. 1A) and analyzed the data as described in Materials and Methods.

We first focused on the different gene expression profiles in fibroblasts from controls and subjects with HGPS that were not treated with FTI. We found that 352 genes were significantly differentially expressed between fibroblasts from subjects with HGPS and controls. Of those genes, 306 were downregulated and 46 upregulated in fibroblasts from subjects with HGPS. The assigned subcellular localizations indicated that at least 31.6% of the gene products were localized to the nucleus, 30.4% to the cytoplasm, 23.7% to the plasma membrane and 1.8% to the extracellular matrix, with the remainder unknown (Fig. 1B). Molecular function analyses (Fig. 1C) and physiological distributions (Fig. 1D) indicated that a significant number of genes were implicated in lipid metabolism, cell growth and differentiation, cell cycle, DNA replication and repair as well as cardiovascular system development.

**Figure 2:**
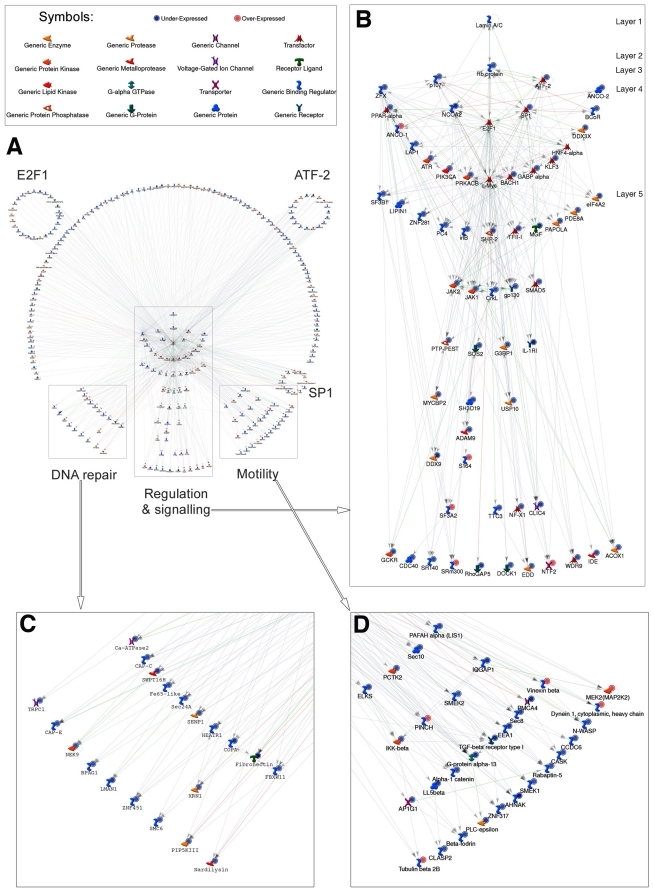
The lamin A-Rb network. The main network (center left) shows downstream interactions between lamin A/C and the 352 differentially expressed genes in HGPS fibroblasts. The network was built using MetaCore analyses, which finds known interactions between gene products. (A) The network divides into several distinct regions, the main region being the regulatory and signaling network. (B) The detailed view of the main region (top right) shows that the only gene differentially expressed in HGPS directly downstream of lamin A/C (layer 1) is that encoding Rb (layer 2). In turn, most of the immediate downstream partners of Rb included in this dataset are p107, NCOA2, SP1, and ATF-2 and E2F1 (layer 3). Of the remaining genes that occur downstream of layer 3, nearly half of the 280 genes interact with only one or more of these transfactors associated to the main network (center left); these gene products are placed above the centre. From layer 4 and 5, most of the genes can be connected at least to one entity according to GeneGO. In the HGPS dataset, 124 genes that had no known interactions in GeneGO are not shown. From the center regulatory and signaling network (zoom left, panel B)), several groups of genes segregate into six subnetworks, based on mutual interactions: a group denoted DNA repair (bottom left, panel (C)), motility (bottom right, panel (D)), and three circles of genes regulated by E2F1, ATF-2 and SP1, respectively. Symbols associating the genes with functions are indicated. Genes labeled with blue circles were downregulated and red circles were upregulated. Higher magnifications of panels A to D are provided in Figures S1, S2, S3 and S4.

Of the 352 genes differentially expressed in cells from subjects with HGPS, 280 genes had known interactions according to MetaCore database (www.genego.com). To build networks integrating these genes, we added *LMNA* because, although its levels of expression remained unchanged, mutations in this gene, which result in abnormal protein expression, are the cause of HGPS (Fig. 2). Of the genes with altered expression in HGPS, the MetaCore method identified *Rb1* as the only one encoding a protein product, Rb, known to interact directly with A-type lamins [29], [30]. The expression of *Rb1* was downregulated in HGPS. Based on differential expression of other genes and known direct protein interactions and relationships, the MetaCore analysis identified additional downstream factors in a signaling network that were altered in cells from subjects with HGPS (Fig. 2). This signaling network started with lamin A/C (layer 1), which is altered by the *LMNA* mutation leading to progerin expression, and impacted Rb (layer 2). The most direct downstream targets of Rb1 were p107, NCOA2, SP1 and ATF2 (layer 3). We added E2F1 to this network, a direct Rb interacting partner although its expression level was not changed. These five factors then had downstream direct effects on 15 other transcription regulators (layer 4). Of these 15 genes, the expression levels of 13 were significantly downregulated in fibroblasts from subjects with HGPS with the exception of ANCO-1, which was upregulated. HNF4-alpha and c-Myc were added to the network even though their levels remained unchanged to allow the integration of the remaining downstream target genes. In the next downstream layer (layer 5), more nuclear factors were connected and all these genes had significantly lower levels of expression in fibroblasts from subjects with HGPS. From the 15 nuclear factors in layer 4, the network divided into several sub-networks, which included the JAK pathway implicated in the regulation of cell proliferation, differentiation and survival, a group of genes related to motility and cytoskeletal organization, and another group of genes implicated in DNA replication and repair (Fig. 2, and Figures S1, S2, S3, S4).

The differential expression of most of the identified genes in HGPS can potentially be explained by the hypothesis that an abnormal prelamin A variant causes Rb to differentially interact with or regulate downstream partners. Most of these genes have at least one direct connection to an upstream nuclear factor (Fig. 2, right panel). Importantly, the differentially expressed genes in HGPS indicated that Rb is a key regulatory component affected by *LMNA* mutation and that it is at the center of a signaling network that is abnormally active in the disease.

### The lamin A-Rb signaling network is modulated by FTI treatment

**Figure 3:**
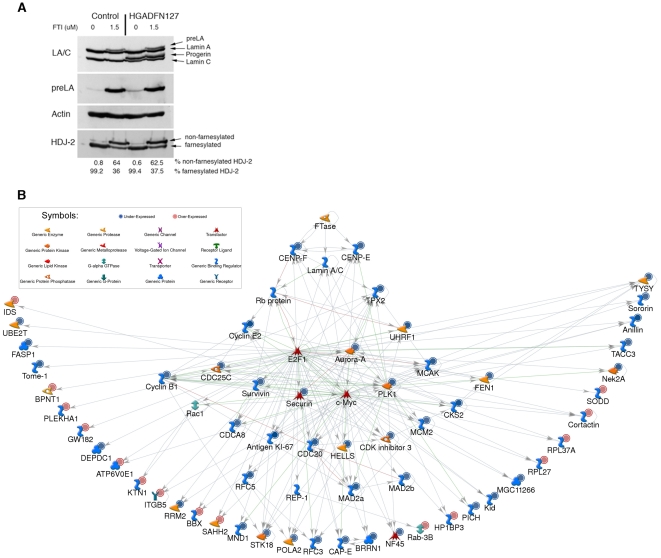
FTI inhibition of prelamin A and progerin farnesylation and FTI-induced gene expression changes in fibroblasts from normal subjects. (A) Western blot analysis of protein extracts of fibroblasts from individuals with HGPS and controls that were treated or untreated with FTI (1.5 µM lonafarnib daily for three days). Blots were probed with anti-lamin A/C (LA/C), anti-prelamin A (preLA), anti-actin, and anti HDJ-2 antibodies. Increase in levels of prelamin A and non-farnesylated HDJ-2 with FTI treatment are indicated. (B) 65 genes were differentially expressed in FTI-treated control cells. The signaling network was built upon functional association between protein farnesyltransferase (FTase), the enzymatic activity of which is inhibited by FTI, lamin A/C and Rb even though expression of all three transcripts remained unchanged with FTI treatment. Of note, lamin A is a known substrate for FTase. Downstream FTase interactions between all the 65 genes differentially expressed in FTI-treated control cells have been incorporated based on their interactions according to MetaCore analysis. The MetaCore analysis identified two transfactors, E2F1 and c-Myc, and one transfactor regulator, REP-1 that permit linking all those 65 genes into this single network. Symbols associating the genes functions are indicated. Genes labeled with blue circles were downregulated and by red circles were upregulated.

To unravel the mechanism underlying reversal of the HGPS phenotype by blocking protein farnesylation, we determined the gene expression changes occurring in fibroblasts after FTI administration. Fibroblasts from normal subjects and subjects with HGPS were grown in medium supplemented daily with 1.5 µM of lonafarnib for three days. Trypan blue exclusion assays indicated that less than 2% of cells died whether or not they were treated with FTI (data not shown). We monitored the inhibition of protein farnesyltransferase by screening for unprocessed HDJ-2 and prelamin A. FTI-treated control cells exhibited an average of 64.0%±0.7 (mean ± SD., n = 3, P<0.05) non-farnesylated HDJ-2 and accumulation of prelamin A (Fig. 3A). FTI-treated fibroblasts from subjects with HGPS exhibited 62.3%±1.5 (n = 3, P<0.05) non-farnesylated HDJ-2 with a similar increase in prelamin A (Fig. 3A), indicating that protein farnesyltransferase activity was similarly inhibited in cells from control individuals and those with HGPS.

We first examined the effects of FTI treatment on normal fibroblasts by using Ingenuity Pathways Analysis (IPA) to compare gene expression profiles in fibroblasts from control individuals with or without FTI treatment. This analysis identified significant differences in the expression of 65 genes, with 47 downregulated and 18 upregulated in cells treated FTI. The majority of these genes were functionally assigned to cell cycle, DNA replication and repair and purine and pyrimidine metabolism; 63% of the encoded proteins were nuclear (Figure S5).

The 65 differentially expressed genes were assembled into a network based on known protein interactions in the MetaCore database. The changes in FTI-treated control cells can be explained by a direct effect of inhibition of protein farnesyltransferase (Fig. 3B). Inhibition of protein farnesyltransferase would subsequently affect three of its substrates, centromeric proteins F and E (CENP-F and CENP-E), the genes of which had altered expression in FTI-treated cells, and lamin A/C. Even though lamin A/C levels remained unchanged it was added to the network because FTIs induce the accumulation of unfarnesylated, unprocessed prelamin A. CENP-E interacts with TPX2 and CENP-F interacts directly with Rb. These proteins therefore were added to the network even though their levels remained unchanged in FTI-treated cells. Rb directly interacts with lamin A/C and with E2F1, repressing its transcriptional activity. Expression of all of the downstream targets of E2F1 was downregulated in the FTI-treated cells (Fig. 3B). These results indicate that FTI treatment affects a sequential cascade of events starting from inhibition of protein farnesyltranferase, which in turn modifies centromeric proteins and lamin A/C and possibly their interactions with Rb. Alterations in Rb function in turn influence the activity of the transcription factor E2F1, a key regulator of downstream genes with decreased expression in fibroblasts after FTI treatment.

### FTI restores a nearly normal gene expression profile in fibroblasts from subjects with HGPS

**Figure 4:**
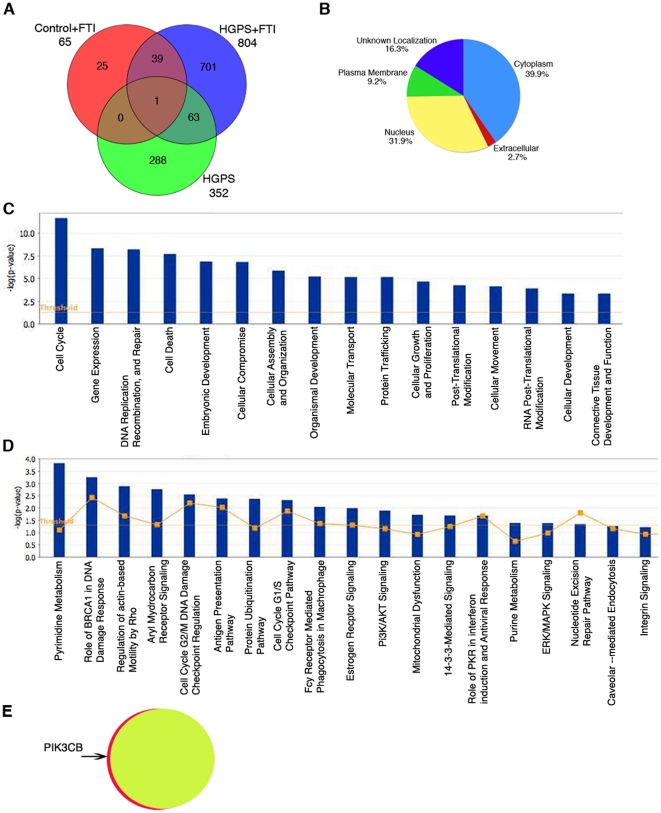
Genome-wide expression profiling of FTI-treated and untreated fibroblasts from subjects with HGPS. (A) Venn diagram comparison of microarray datasets of controls versus control-FTI, or HGPS and/or HGPS-FTI. 65 genes were differentially expressed in fibroblasts from control subjects after FTI treatment compared to no treatment (Control+FTI; red); 352 in fibroblasts from subjects with HGPS compared to those from control subjects (HGPS; green); and 804 in fibroblasts from subjects with HGPS after FTI treatment compared to untreated controls (HGPS+FTI; blue). Genes with altered expression common between these different datasets are shown as areas of overlap with different colors in the Venn diagram with numbers of common genes indicated. (B) Pie chart indicates the subcellular localization of the encoded proteins of the differentially expressed genes between control and HGPS-FTI datasets according to information contained in the Ingenuity Knowledge Base. (C) Genes differentially expressed between controls versus HGPS-FTI datasets were assigned to diverse cellular functions according to IPA and (D) were associated to canonical pathways according to IPA. The significant genes were compared to IPA database and ranked according to p-values generated with Fisher extract test. P-values less than 0.005 indicate a statistically significant, non-random association between a set of significant genes and a set of all genes related to a given function in IPA database. The ratio (y-axis) represents the number of all known genes ascribed to the pathway. The Yellow line represents the threshold of p<0.05. (E) Comparison of gene expression alterations between FTI-treated control cells (yellow) with FTI-treated fibroblasts from subjects with HGPS (red). Using criteria of corrected p-value from unpaired t-test <0.01 and two-fold change in expression, only one gene, *PIK3CB* was upregulated in FTI treated cells from subjects with HGPS compared to cells from normal subjects treated with FTI. Therefore, 99% of the genes in FTI-treated cells from subjects with HGPS were expressed at the same levels as in FTI-treated normal fibroblast.

To determine the effects of a FTI on gene expression in cells from subjects with HGPS, we generated a Venn diagram to demonstrate overlapping alterations in expression in three datasets (Fig. 4A). The three datasets were: (1) genes differentially expressed in fibroblasts from controls subjects after FTI treatment compared to no treatment (65 genes); (2) genes differentially expressed in fibroblasts from subjects with HGPS compared to those from control subjects (352 genes); and (3) genes differentially expressed in fibroblasts from subjects with HGPS after FTI treatment compared to those from untreated control subjects (804 genes). Differential expression of 25 genes were specific to FTI-treated control fibroblasts (dataset 1); 40 genes were commonly differentially expressed in these cells as well as in cells from subjects with HGPS treated with FTI (dataset 1 compared to dataset 2). Only one gene, *SMC2*, a structural maintenance factor of chromosome 2 was common between the three datasets.

We focused on the gene expression profile resulting from FTI treatment of fibroblasts from subjects with HGPS (dataset 3), which indicated changes in the expression of 804 genes after FTI treatment. Of these 804 genes, 414 were upregulated and 390 were downregulated. This high number of genes with altered expression suggests that cells from subjects with HGPS were highly sensitive to FTI. For the gene products, 31.9% were predicted to be nuclear proteins (Fig. 4B), whereas in normal fibroblasts treated with FTI 63% of the differentially expressed genes were predicted to encode nuclear proteins (Figure S5). Of these 804 genes showing differential expression after FTI treatment of cells from subjects with HGPS, 64 were differentially expressed in untreated cells from subjects with HGPS compared to normal fibroblasts. Expression of the remaining 701 differentially expressed genes were uniquely altered in FTI-treated fibroblasts from subjects with HGPS and were functionally associated with gene expression, cell cycle, cell growth, DNA replication and repair, molecular transport and protein trafficking (Fig. 4C). They also were involved in several canonical pathways involved in PI3K/AKT signaling, protein ubiquitination and regulation of the actin cytoskeletal network (Fig. 4D).

Only 64 genes were differentially expressed in both the datasets comparing fibroblasts from control to HGPS (dataset 2) and comparing fibroblasts from control to HGPS treated with FTI (dataset 3). Hence, abnormal expression of 288 of the 352 genes in fibroblasts from subjects with HGPS was normalized after FTI-treatment.

In addition, an additional t-test analysis was done directly comparing fibroblasts from control individuals treated with FTI to fibroblasts from subjects with HGPS treated with FTI. This analysis indicated that only 1 gene *PIK3CB* (+2.166) was altered in FTI-treated HGPS cells (Fig 4E). *PIK3CB* is a phosphoinoitide-3-kinase that is functionally associated to apoptosis, survival, migration, proliferation, cell cycle and adhesion. The IPA analysis links *PIK3CB* with one network associated to lipid metabolism. Therefore, the difference in expression of only one gene indicated that 99% of the genes in FTI-treated cells from subjects with HGPS were expressed at the same levels as in FTI-treated normal fibroblasts.

### Validation of the microarray analyses by real-time RT-PCR

**Figure 5:**
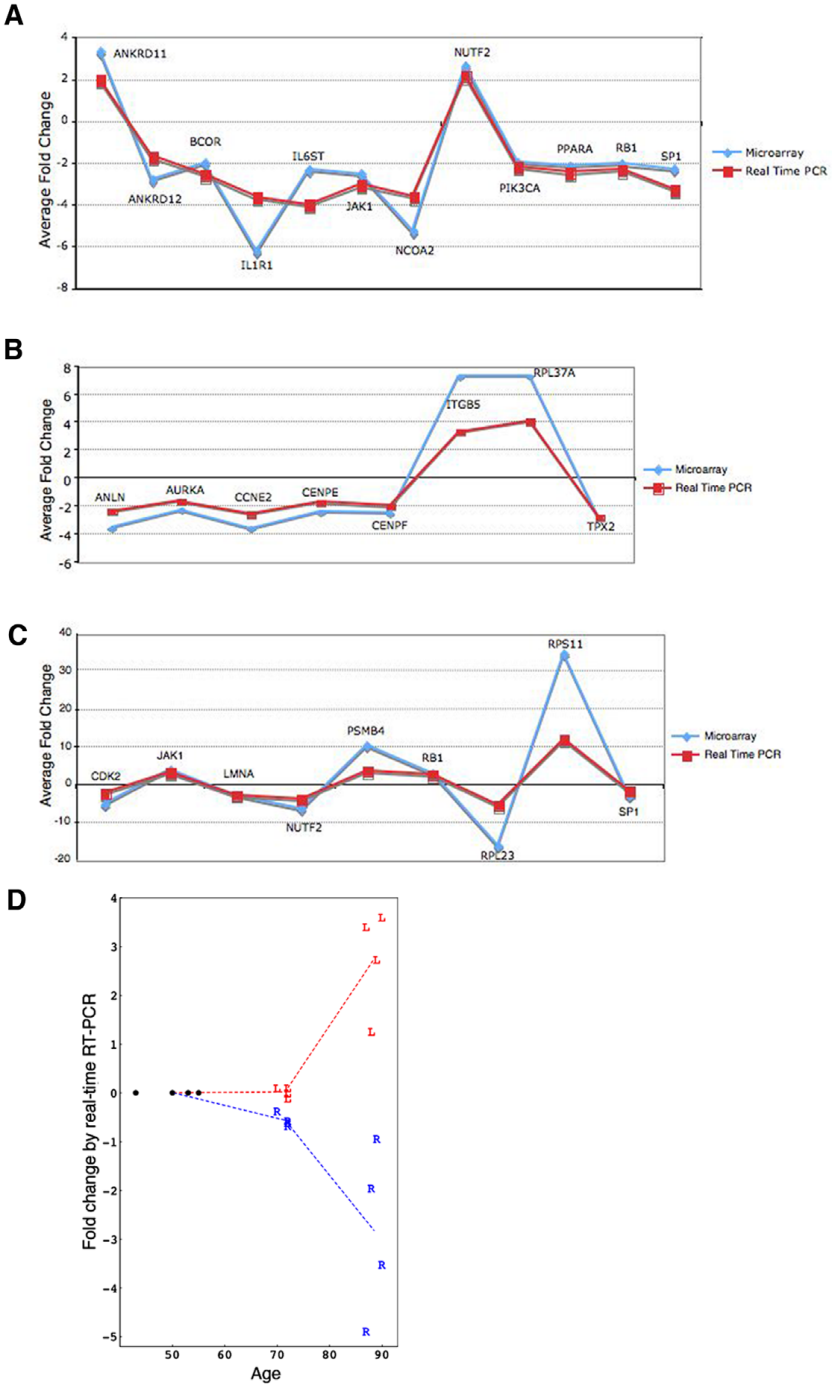
Real-time RT-PCR validation of microarray results for selected genes. (A) Validation of a set of genes identified in microarray analysis comparing fibroblast from subjects with HGPS to controls. The mean value of expression for indicated genes measured using real time PCR (red; *p*<0.05), and microarray (blue; *p*<0.01) are shown. (B) Validation of a set of genes identified in microarray analysis comparing fibroblasts from control subjects with and without FTI treatment. (C) Validation of a set of genes identified in microarray analysis comparing fibroblasts from subjects with HGPS with and without FTI treatment. (D) Relative expression of *LMNA* (L, red) and *RB1* (R, blue) in human skin derived from 16 unaffected individuals of various age groups was analyzed by quantitative real-time RT-PCR. A relative quantification method was used and the data from the 38 to 55-year-old age group were calibrated to a relative quantity of 1. All values were normalized to GAPDH (Materials and Methods).

Differential expression of selected genes found to be significantly different in the microarray analyses was confirmed by real-time RT-PCR. To compare fibroblasts from subjects with HGPS to unaffected controls, we selected genes identified in the microarray analysis that were upstream regulators in the signaling pathway. For normal fibroblasts with and without FTI treatment, we selected genes that were upstream regulators in the signaling network. For fibroblasts from subjects with HGPS with and without FTI treatment, we selected genes that showed the greatest fold changes in expression. We also measured the expression of PIK3CB in FTI-treated fibroblasts from subjects with HGPS and unaffected controls, as this was the only gene identified by microarray analyses to be differentially expressed between these experimental groups. In all cases, gene expression differences measured by real-time RT-PCR were consistent with those measured in the microarray analyses (Fig. 5A, B, C and Figure S6).

### Potential relationship between gene expression alterations in HGPS and physiological aging

**Figure 6:**
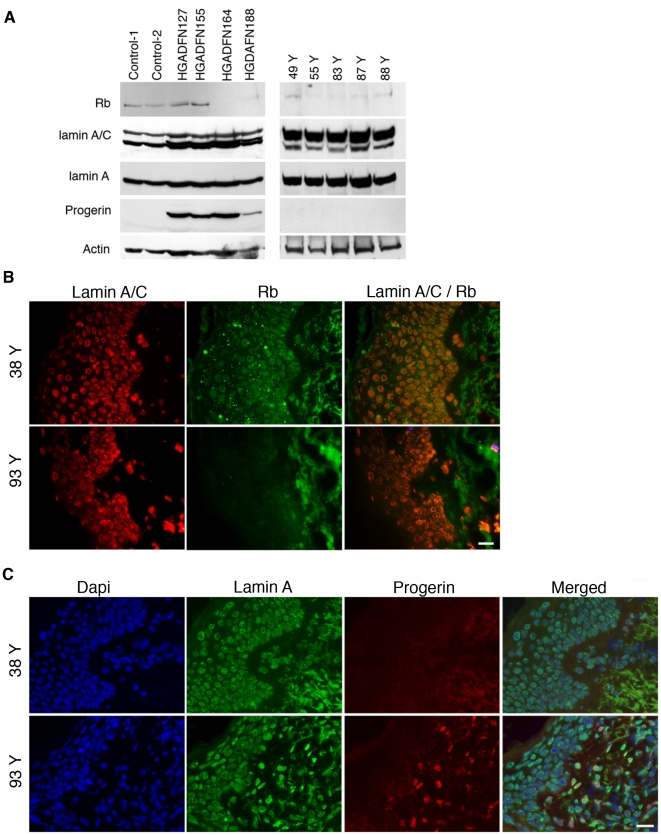
Detection of lamin A/C and Rb in cells from subjects with HGPS and in normal human skin biopsies. (A) Left panel: Western blot of proteins extracted from control fibroblasts (Control-1 [GM02036A], Control-2 [GM03349C]) and fibroblasts from subjects with HGPS (HGADFN127, HGADFN155, HGADFN164, HGADFN188) sequentially probed with anti-lamin A/C, anti- lamin A, anti-progerin (progerin) and anti-actin antibodies. Right panel: Western blot of protein extracts from skin of individuals at different ages as indicated (49 to 88 Year old, Y denote year). Blots were sequentially probed with antibodies cited above. (B) In situ detection of lamin A/C and Rb in human skin sections. Skin sections from 93-year-old and 38-year-old subjects were probed with antibodies against lamin A/C (red) and Rb (green) and signal overlap is shown at right (Lamin A/C/Rb). (C) Skin sections as in panel (B) were probed with antibodies against lamin A (green) and progerin (red) and DNA was stained with 4′,6-diamidino-2-phenylindole (blue); the merged signals are indicated. Scale bar, 20 µM.

Our results implicate a defective lamin A-Rb signaling network as a pathogenic mechanism in HGPS, a disease with features of premature aging. We investigated whether alterations in this same signaling network might occur during physiological aging. We screened mRNA extracted from human skin biopsies from individuals 38 to 90 years of age. The skin samples were derived from an already established skin biopsy tissue bank (approved by the Columbia University Medical Center Institutional Review Board), which has been described previously [31]. Total mRNA extractions from human skin samples were performed as described previously [31]. Remarkably, lamin A/C transcripts were increased in skin from individuals 87 to 90 years old (Fig. 5D). In contrast, Rb mRNA was already decreased in skin from 70 to 72-year-old subjects (Fig. 5D), indicating that changes in *LMNA* and *RB1* expression occur during physiological aging. Furthermore, Western blot analyses of proteins extracted from skin showed that A-type lamins were increased in samples from middle aged and elderly individuals (Fig. 6A, B). Low levels of progerin were also detected by indirect immunofluorescence microscopy using a specific antibody in a restricted number of dermal fibroblasts in skin sections derived from elderly subjects (Fig. 6C). However, progerin expression was too low to detect by Western blot analyses of proteins extracted from skin as previously reported [31]. Rb, which is expressed at low levels in normal skin, was weakly detected using immunofluorescence microscopy in samples from young individuals and barely detected in samples from older subjects (Fig. 6A left panel). These findings indicate that lamin A and Rb levels change during physiological aging and suggest that defects in a lamin A-Rb signaling pathway might occur similarly to what we have identified in cells from subjects with HGPS.

## Discussion

Inhibition of protein farnesyltransferase activity by FTIs has been shown to improve abnormalities in nuclear morphology in cells from subjects with HGPS and from animal models of the disease [15], [16], [17], [18], [19], [20], [32]. FTIs also improve the progeria-like phenotypes of mouse models of HGPS [22], [23]. Presumably, blocking farnesylation of progerin, the truncated prelamin A expressed as a result of the G608G *LMNA* mutation which is responsible for most cases of HGPS, renders it less toxic. However, the molecular mechanisms by which progerin exerts its toxicity and whether FTI treatment reverses specific molecular defects induced by progerin remain largely unknown. We have now identified a defective lamin A-Rb signaling network in cells from subjects with HGPS and demonstrated that treatment of cells with an FTI reverses abnormalities in the expression of genes encoding proteins in this network.

We compared our gene expression profiles to those of previous studies that have examined gene expression in HGPS and found very little overlap (3.5% overlap of differentially expressed genes with Scaffidi and Mitseli [28] and less than 1% with Csoka *et al.* [27] (Table S1). Csoka *et al.* [27] used fibroblasts from only three subjects ages 8 to 14 years of age whereas we used cultured fibroblasts from five subjects with HGPS obtained at earlier ages (age 1 to 4 years). As the growth rate and proliferation potency of fibroblasts from subjects with HGPS decrease with cellular age *in vitro* and the donor's age, this could partially explain the differences between our results. Importantly, the average age of death in HGPS is 12–15 years, which suggests that the cellular phenotype may be severely altered in older children [4]. Scaffidi and Misteli [28] generated immortalized normal fibroblasts that overexpressed progerin and examined gene expression in only two lines, making a comparison difficult given the very different system and the low statistical power from using only two biological replicates. Separately, Fong et al. [33] used RT-PCR to examine expression of several genes selected from these previous studies in normal fibroblasts transfected with antisense oligonucleotides that increase *LMNA* alternative RNA splicing to generate progerin. We found that four of seven genes they tested were similarly differentially expressed in the fibroblasts we used from subjects with HGPS (Table S2). An earlier study by Ly *et al*. [25] used fibroblasts from three donors with HGPS of age 8 to 9 years and found 61 genes differently expressed in young subjects compared to old subjects and with HGPS subjects. Of these 61 genes, 49 were differentially expressed in HGPS versus young individuals [25]. Comparison of these 49 genes against the 352 genes differentially expressed in HGPS identified in our study indicated an overlap of 22 genes. An additional study from Park *et al* [26] compared gene expression in fibroblasts from only one subject with HGPS to that of cultured fibroblasts in replicative senescence; no statistical criteria were applied. Although HGPS is an extremely rare disease, to the best of our knowledge we have used the most biological replicates so far in any study of genome-wide gene expression in actual patient material. We also validated our microarray data using information from the literature, by screening databases and by performing RT-PCR for selected genes. We confirmed by RT-PCR that Rb mRNA levels were decreased in HGPS cells in comparison the control cells. No change in Rb gene expression level was reported in previous microarray analyses on HGPS cells or HGPS cell models [26], [27], [28].

Most importantly, rather than providing a descriptive list of genes with altered expression levels, our methods identified a novel signaling network starting with the lamin A-Rb interaction that is altered in HGPS.

The potential link between progerin and Rb in the orchestration of cell cycle and proliferation defects was previously suggested by studies demonstrating that lamin A and C interact with Rb [30], [34]. Rb is a tumor suppressor and major cell cycle regulator that, in its hypophosphorylated state, binds to and inhibits the E2 factor (E2F) family of transcription factors required for cell cycle progression [36]. Upon hyperphosphorylation of Rb by cyclin/cyclin-dependent kinase complexes, E2F is released to initiate S phase. A role for lamins in this process is further suggested by the finding that hypophosphorylated Rb is tightly associated with lamin A/C-enriched nucleoskeletal preparations of early G1 cells [29]. In HGPS cells there is evidence for a significant reduction in hyperphosphorylated Rb in HGPS fibroblasts (Dechat et al. 2007). In our experiments, we found a decreased level of Rb expression at the mRNA and protein levels in HGPS (see Figure 2 and Figure 6).

Previously, a small fraction of lamin A/C was shown to colocalize with Rb and E2F1 in nuclear foci during G1-phase [37]. In HGPS cells, we could not detect lamin A foci. Whether Rb foci remain associated with E2F1 remains to be further investigated. In HGPS cells, our microarray analysis indicated a decrease in Rb levels without changes in E2F1 levels. We further investigated whether E2F1 levels might be altered by indirect immunofluorescence microscope and observed no changes in the nuclear intensity of the E2F1 signal between HGPS and normal fibroblast cells (data not shown). Previously, the localization of Rb and E2F1 in cells from *Lmna* ^−/−^ mice indicated that while Rb levels were reduced, a portion of Rb remained localized to nuclear foci but displayed reduced overlap with the E2F1 signal [35]. The expression levels of E2F1 *in Lmna* ^−/−^ cells in comparison to wild type cells has not yet been reported. However, the lack of association between Rb and E2F1 in nuclear foci in the absence of lamin A/C could indicate an alteration in Rb control of E2F1 activity [35]. Further studies will be needed to understand how expression levels of lamin A/C, Rb, and E2F1 might affect each other's expression levels, interactions and subnuclear localization. In addition to its role in cell cycle control, Rb also regulates cellular differentiation. In the absence of lamin A/C in muscle cells derived from *Lmna* ^−/−^ mice, reduced levels of Rb and other transcription factors regulating muscle cell differentiation was reported [38]. This study provide an additional link between lamin A/C-Rb and its potential role in muscle cell differentiation [38].

Immunohistochemical analysis of Rb distribution did not reveal any obvious alteration besides a decreased Rb signal in some nuclei of cells from subjects with HGPS (Figure S7). Rb was also detected in the most dysmorphic nuclei exhibiting a strong progerin staining, indicating the accumulation of high levels of progerin protein (Figure S7) as reported previously [15]. Based on these previous studies, decreased Rb expression or phosphorylation status in HGPS cells appears to be implicated in the deregulation of proliferation. Whether the interaction of A-type lamin with Rb is impaired in HGPS cells remains to be further addressed. However, our present study together with previously published observations support the potential implication of a defective lamin A/C-Rb signaling network in the HGPS cellular phenotype. In support of our finding, a similar potential link between an altered Lamin A/C-Rb signaling was previously reported in cells derived from Lmna^−/−^ mice model [35]. In cells lacking lamin A/C the levels of Rb was decreased while its mRNA level remained unchanged in comparison to wild type cells [35]. These findings suggested that the Rb was unstable in the absence of lamin A/C. In HGPS cells, we observed a decrease in both mRNA and protein levels of Rb. Our findings indicate that the lamin A/C-Rb signaling might be altered differently depending on the status of A-type lamins in cells (absence of A-type lamins expression versus expression of an abnormal lamin A variant, progerin). Further studies are required to understand the molecular mechanisms driven by alterations in A-type lamins on Rb signaling pathways.

FTI treatment of cells from subjects with HGPS leads to a significant reversal of the abnormal expression of genes encoding proteins in the lamin A-Rb signaling network. This is consistent with the improved phenotypes that result from blocking protein farnesylation in cellular and animal models of the disease [16], [17], [18], [20], [21], [23]. Defective Rb activity appeared to be responsible for the repression of the large set of downstream transcription factors and regulators. To evaluate the impact of FTIs, we also investigated FTI-induced gene expression changes in fibroblasts from unaffected controls. Remarkably, Rb appeared again in this dataset as a key regulator of the downstream events. Not surprisingly, FTI-treated cells from subjects with HGPS demonstrated a near total reversal (99%) of abnormally expressed genes in comparison to FTI-treated control cells. Our finding suggests that a potential correction of the HGPS phenotype does not necessarily require a full inhibition of protein farnesyltransferase but rather points to the importance of normalizing Rb expression levels and function.

The results of our study further support the potential use of FTIs as therapeutic agents in HGPS. A phase II clinical trial treating children with HGPS with lonafarnib, the FTI we used in this study, was initiated in spring of 2007 [39]. Because of known toxicity, the dose of lonafarnib administrated to these children may generate tissue concentrations below those necessary to obtain the effects we observed *in vitro*. Therefore, in human subjects, other drugs in addition to an FTI may be necessary to inhibit protein prenylation to a similar extent as in cultured cells. One possibility is to add a statin and an aminobisphosphonate, which have been shown to inhibit prelamin A prenylation and improve the phenotype of mice deficient in Zmpste24 [24]. Furthermore, our findings suggest that screening candidate molecules against targets we identified in the lamin A-Rb signaling network could identify novel therapeutic approaches to treat HGPS. Proteins in this network could also serve as biomarkers for HGPS.

Because the lamin A-Rb interaction is a new signaling axis in the pathogenesis of HGPS, we investigated its potential role in physiological aging. We found that *LMNA* and *RB1* expression appeared to be inversely modulated in the elderly. These observations were similar to our findings in cells from subjects with HGPS. Although lamin A and lamin C levels were not altered, an abnormal prelamin A variant, progerin, is expressed and Rb expression is decreased. In addition to changes in the expression of A-type lamins, several studies suggest that abnormal prelamin A may accumulate in normal aging [14], [31], [40], [41]. Our results, therefore, suggest that therapeutic approaches to re-establish a proper lamin A-Rb signaling network may be beneficial in preventing complications of physiological aging.

## Materials and Methods

### Cell culture and FTI treatment

Dermal fibroblasts from subjects with HGPS were obtained from the Progeria Research Foundation (www.progeriaresearch.org). The following fibroblasts were used: HGADFN003 (M, age 2), HGADFN127 (F, age 3), HGADFN155 (F, age 1), HGADFN164 (F, age 4) and HGADFN188 (F, age 2). Age-matched control dermal fibroblasts were obtained from Coriell Institute for Medical Research (Camden, NJ). The following cell lines were used: GM01652C (F, age 11), GM02036A (F, age 11), GM03349C (M, age 10), GM03348E (M, age 10), GM08398A (M, age 8). The Institutional Review Board at Columbia University Medical Center approved the use of human cells established from skin biopsies from patients with HGPS and unaffected individuals.

Cells were cultured in DMEM containing 15% fetal bovine serum, 1% glutamine and 1% penicillin/streptomycin. Cells were subcultured at 80% confluency to keep cultures in growth phase and collected at population doublings between 22 and 27.

Treatment with the FTI lonafarnib (Schering-Plough, Kennilworth, NJ) was initiated when the cells reached 45–50% confluency. Lonafarnib was added to the culture media to a concentration of 1.5 µM FTI daily for three days. Untreated fibroblasts were cultured in parallel with added vehicle (DMSO). Cellular toxicity was determined by Trypan blue exclusion. Preliminary studies were conducted with varying lonafarnib concentrations and 1.5 µM was selected, as higher concentrations caused increased cell death and lower concentrations less effectively blocked protein farnesylation (data not shown).

### RNA Preparation

Total RNA was extracted from the cell pellets using the Rnase Mini kit (Qiagen, Valencia, CA). Spectrophotometric quantification of RNA was performed and the purity assessed by measurement of the 260 nm/280 nm absorbance ratio. Ratios between 1.9 to 2.1 were accepted. Additionally, samples were run on a 1.2% agarose gel to examine integrity of the RNA. All gels showed two discrete bands at the 4.5 kb and 1.9 kb, representing the 28S and 18S ribosomal subunits, respectively (data not shown).

### Microarray processing

RNA samples were amplified once using the One Cycle Target Labeling and Control Reagents and labeled for hybridization using the Affymetrix reagents and protocols (Affymetrix, Santa Clara, CA). Prior to hybridization, biotin-labeled cRNA samples were examined on an Agilent 2100 Bioanalyzer (Agilent Technoligies, Palo Alto, CA) to ensure quality. The samples were hybridized to U133 Plus 2.0 Arrays (Affymetrix) at the Columbia University Microarray Core Facility.

The microarray data have been deposited in the European Bioinformatics Institute's ArrayExpress (www.ebi.ac.uk/arrayexpress) under accession number: E-MEXP-2597 along with detailed protocol notes.

### Microarray data analysis

Data outputs were normalized and analyzed using GeneSpring GX 10.0 commercial software package (Agilent Technologies). Affymetrix data were uploaded into GeneSpring GX, normalized and quality control assessment was run to ensure the integrity of the samples. A t-test comparison was performed for each experimental group. The P-value cutoff was set to 0.01, a Benjamini-Hochberg correction was applied and the significant fold difference was considered two-fold above or below baseline. For interpretation of the results, MetaCore and GeneGo (www.Genego.com) and Ingenuity Pathways Analysis (Ingenuity Systems, www.ingenuity.com) software were used. Datasets containing gene identifiers and corresponding expression values were uploaded in the applications. An up or down change of two-fold or greater was set to identify genes whose expression was differentially regulated. These genes, or focus molecules, were overlaid onto a global molecular network developed from information contained in the Ingenuity Pathways Knowledge Base.

### Network Analysis and Visualization

Gene sets were analyzed using MetaCore (www.genego.com). We built networks using as far as possible all and only genes belonging to the differentially regulated sets. The assembly of the networks exhibited in Fig. 2 and Fig. 3 were initially loaded with only the differentially regulated genes from the respective datasets. Then we added the known causal genes (encoding A-type lamins for Fig. 2 and protein farnesyltransferase for Fig. 3) and manually laid out the remaining genes in a semi-circular network based on the directness of the downstream interactions to the causal gene. In both cases, only a small number of genes had direct downstream interactions. For these and subsequent layers, we applied two rules. Rule 1: if a gene was downstream only to genes on the current layer or more inner layers, and if the gene had no genes downstream of it, it was moved to the outermost layer; otherwise, it was moved to the next layer. Rule 2: after the second layer, if the gene did not have downstream connections to genes in the current or previous layer, the gene was moved to a layer beyond the current layer. Following the initial manual layout, a number of differentially expressed genes were still not assigned to a downstream position from the causal gene. We used the MetaCore transfactor analysis tool to determine which transfactors could be implicated based on published data. This tool produces a list of transfactors belonging to the dataset analyzed. The list is ranked by an estimate of likelihood of being involved. Starting with the most likely, we added transfactors to the network, manually re-adjusting the network each time. We moved down the list until we had either exhausted the possible transfactors or finished the list of genes.

### Real-time RT-PCR analysis

We synthesized cDNA using Omniscript Reverse Transcriptase (Qiagen) using total cellular RNA as template. RNA from fibroblasts from three subjects with HGPS and three controls, cultured with or without FTI, was used. Primers were designed using Primer3 (http://frodo.wi.mit.edu/cgi-bin/primer3/primer3_www.cgi). The list of genes that were validated by RT-PCR and their corresponding primers are shown in Table S3.

Real-time RT-PCR reactions contained Power SYBR Green PCR mastermix (Applied Biosystems), 200 nM of each primer, and 0.1 µl of template in a 20-µl reaction volume. Amplification was carried out using the 7300 Real-Time PCR Detection System (Applied Biosystems) with an initial denaturation at 95°C for two minutes followed by 50 cycles at 95°C for 30 seconds and 62°C for 30 seconds. Three experiments were performed for each assay, in which the samples were run in triplicate. GAPDH was used as an endogenous control and quantification was performed using the relative quantification method where the real-time PCR signal of the experimental RNA was measured in relation to the signal of the control. The 2(ΔΔC_T_) method was used to calculate relative changes in gene expression [42].

### Western blot analysis

Cells were extracted in Laemmli sample buffer (Bio-Rad) and heated for five minutes at 95°C. Approximatey 30 µg total protein extracts were loaded in parallel on a 7.5% polyacrylamide gel. An average of 7 mg of human skin tissues were extracted in Laemmli buffer using the bullet blender according to manufacturer instructions (Next Advance, Averill Park, NY). Approximately 40 µg skin protein extracts were loaded on the gel. After separation by electrophoresis, proteins were transferred to nitrocellulose membranes and incubated with blocking buffer as described previously [12]. Membranes were incubated sequentially with anti-prelamin A C20 antibodies (Santa Cruz Biotechnology, Santa Cruz, CA), anti-lamin A/C antibodies [43] (kindly provided by Dr. Nilabh Chaudhary), anti-actin antibodies (Sigma-Adrich, St. Louis, MO) and anti-HDJ-2 (Abcam, Cambridge, MA), washed and then incubated with a corresponding secondary antibody coupled to horseradish peroxidase (Jackson ImmunoResearch Laboratories, West Grove, PA). Other blots were similarly incubated sequentially with anti-Rb (BD Biosciences Pharmingen, San Diego, CA), anti- progerin [31], anti-lamin A (133A2, abcam), anti-laminA/C and anti-actin antibodies. Proteins were visualized using the enhanced chemiluminescence detection system (GE Healthcare, Piscataway, NJ). Signals obtained on the autoradiograms were analyzed by densitometry using Quantity One 1-D analysis software (Bio-Rad) on the scanned images.

### Indirect immunofluorescence microscopy

Immunohistochemistry was performed on 6 µm frozen human skin sections fixed in methanol/acetone (1V/1V) at −20°C for 10 minutes and washed in phosphate-buffered saline, then blocked in phosphate-buffered saline containing 3% bovine serum albumin, 10% normal goat serum and 0.3% Triton X-100 for 30 minutes and 1 hour in the same buffer without Triton X-100. Cells and slides were incubated with anti-laminA/C and anti-Rb (BD Biosciences Pharmingen), or anti-progerin and anti-Rb or anti- lamin antibodies. The secondary antibodies were affinity purified Alexa Fluor 488 goat or donkey immunoglobulin G antibodies (Life Technologies, Carlsbad, CA) and Cy3-conjugated IgG antibodies (Jackson ImmunoResearch laboratories). All samples were also counterstained with 4′,6-diamidino-2-phenylindole (Sigma-Aldrich). Images were acquired on an Axioplan fluorescence microscope (Carl Zeiss, Thornwood, NY).

## Supporting Information

Figure S1 High magnification of Fig. 2, panel A.
(1.37 MB TIF)

Figure S2 High magnification of Fig. 2, panel B.
(3.89 MB TIF)

Figure S3 High magnification Fig. 2, panel C.
(1.40 MB TIF)

Figure S4 High magnification Fig. 2, panel D.
(1.63 MB TIF)

Figure S5 Genome-wide expression profiling of FTI-treated and untreated control fibroblast cultures. (A) Genes differentially expressed in normal fibroblasts treated with FTI compared to untreated normal fibroblasts were assigned to diverse cellular functions according to IPA, and (B) were associated with canonical pathways according to IPA. (C) Pie chart indicates the subcellular localization of the protein products of the differentially expressed genes according to information contained in the Ingenuity Knowledge Base.
(0.37 MB TIF)

Figure S6 Validation of the microarray analysis by real-time RT-PCR. (A) Validation of a set of genes identified in microarray analysis comparing fibroblasts from subjects with HGPS to control. The mean value of expression for indicated genes measured using real time RT-PCR are indicated (SD: standard deviation; p<0.05), and microarray fold change (p<0.01) are shown. (B) Validation of a set of genes identified in microarray analysis comparing fibroblasts from control subjects with and without FTI treatment. (C) Validation of a set of genes identified in microarray analysis comparing fibroblasts from subjects with HGPS treated with FTI to untreated fibroblasts from control subjects. (D) Validation of the only gene differentially expressed between FTI-treated fibroblasts from subjects with HGPS to FTI-treated fibroblasts from control subjects. Fold changes measured by real-time RT-PCR and microarray analyses are indicated.
(0.71 MB TIF)

Figure S7 Distribution of progerin and Rb in cells from a subject with HGPS. Immunohistochemy was performed on fibroblasts from an unaffected control (GM03348) and a subject with HGPS (HGADFN003) at PPD 25 to 30. Cells were stained with anti-progerin antibody [31] (red) and anti-Rb monoclonal antibody (BD Biosciences Pharmingen) (green). Chromatin was stained with dapi. The triple merged signals are indicated. Scale bar, 20 µM.
(1.31 MB TIF)

Table S1 Comparison of the differentially expressed genes in fibroblasts from subjects with HGPS from this study to the differentially expressed genes lists in studies by Scaffidi and Mitseli [28] and Csoka et al.[27] Control versus HGPS differentially expressed genes established after a statistical analysis using the t test with 1% significance and 2-fold cutoff were compared to the initial microarray analyses performed on three HGPS fibroblast strains (Coriell cell repositories) derived from patients at age 8 (AG11513), 9 (AG10750) and 14 years old (AG11498)[27]. This small overlap may be due to variation intrinsic to each cell, in addition to the fact that cells from subjects with HGPS exhibit increased variation in cellular phenotype with cellular age in vitro and with the donor's age. Our study was performed using five fibroblast cultures from five subjects with HGPS at age 2 to 4 years old, kindly provided by Progeria Research Foundation. Cells were collected at an early PPD (<25) when their growth rate remained similar to that of control fibroblast cultures. Phenotypic variations and different levels of progerin expression can contribute to the heterogeneity between fibroblasts from subjects with HGPS. We compared the control versus HGPS gene list with another study from Scaffidi and Mitseli that used a cellular model for HGPS by overexpressing progerin in normal immortalized fibroblasts [28] and found very little overlap.
(0.07 MB TIF)

Table S2 Screening of a set of genes that were previously suggested to be perturbed in HGPS cells [28], [33]. Using the same olignucleotides for RT-PCR described by Fong et al 2009[2], we screened cultured fibroblasts from subjects with HGPS and from control individuals used in this study. The fold change between fibroblasts from subjects with HGPS and from control individuals in mRNA transcript for the corresponding genes are indicated with a P<0.05.
(0.09 MB TIF)

Table S3 List of primers used for real time PCR.
(21.31 MB TIF)
