# Supplementary figures and images for: Defective Lamin A-Rb Signaling in Hutchinson-Gilford Progeria Syndrome and Reversal by Farnesyltransferase Inhibition
Source: PLoS One. 2010 Jun 15;5(6):e11132. doi: 10.1371/journal.pone.0011132 (PMC2886113; doi:10.1371/journal.pone.0011132)

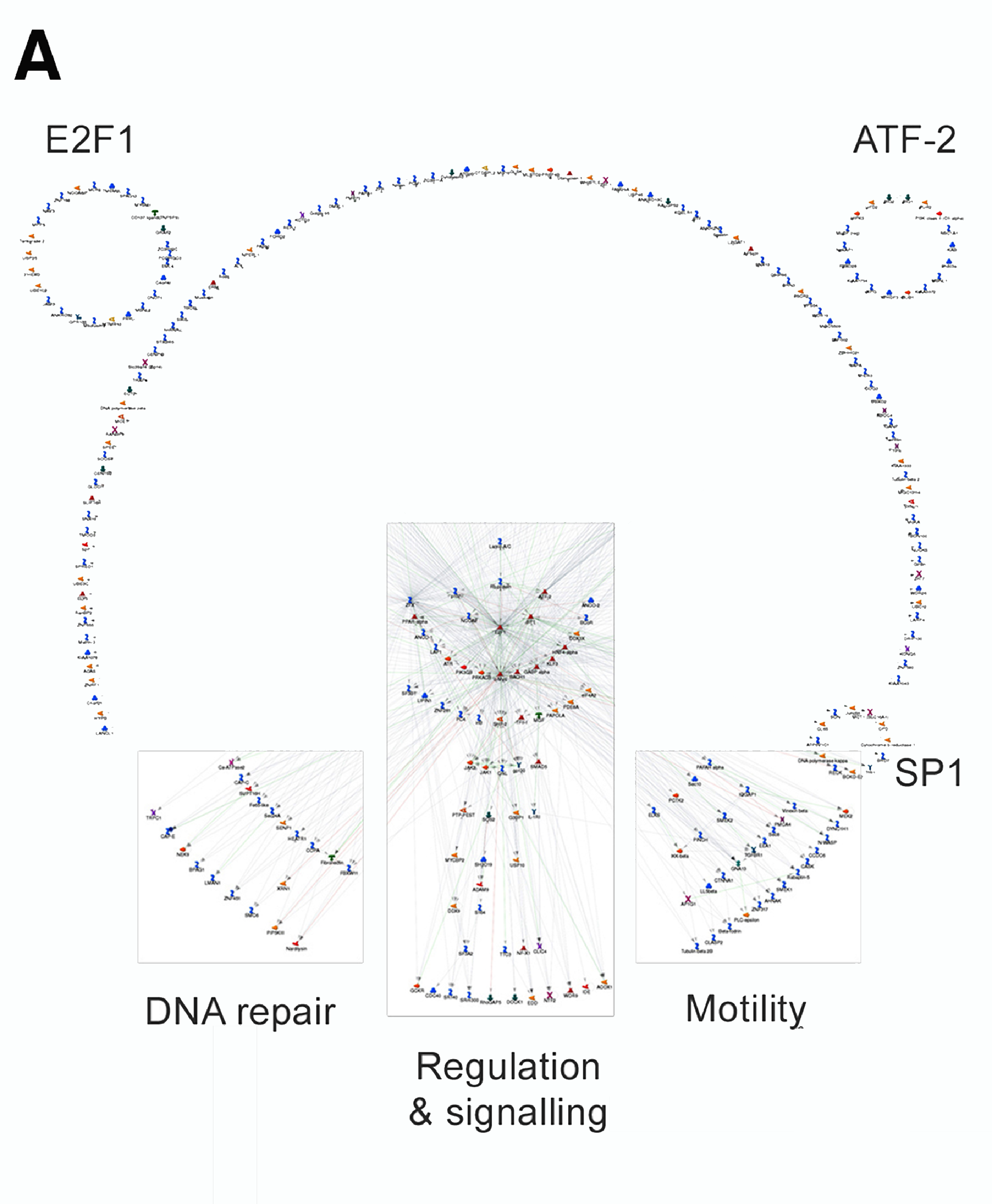

Supplement: Figure S1 — High magnification of Fig. 2, panel A. (1.37 MB TIF) [file pone.0011132.s001.tif]

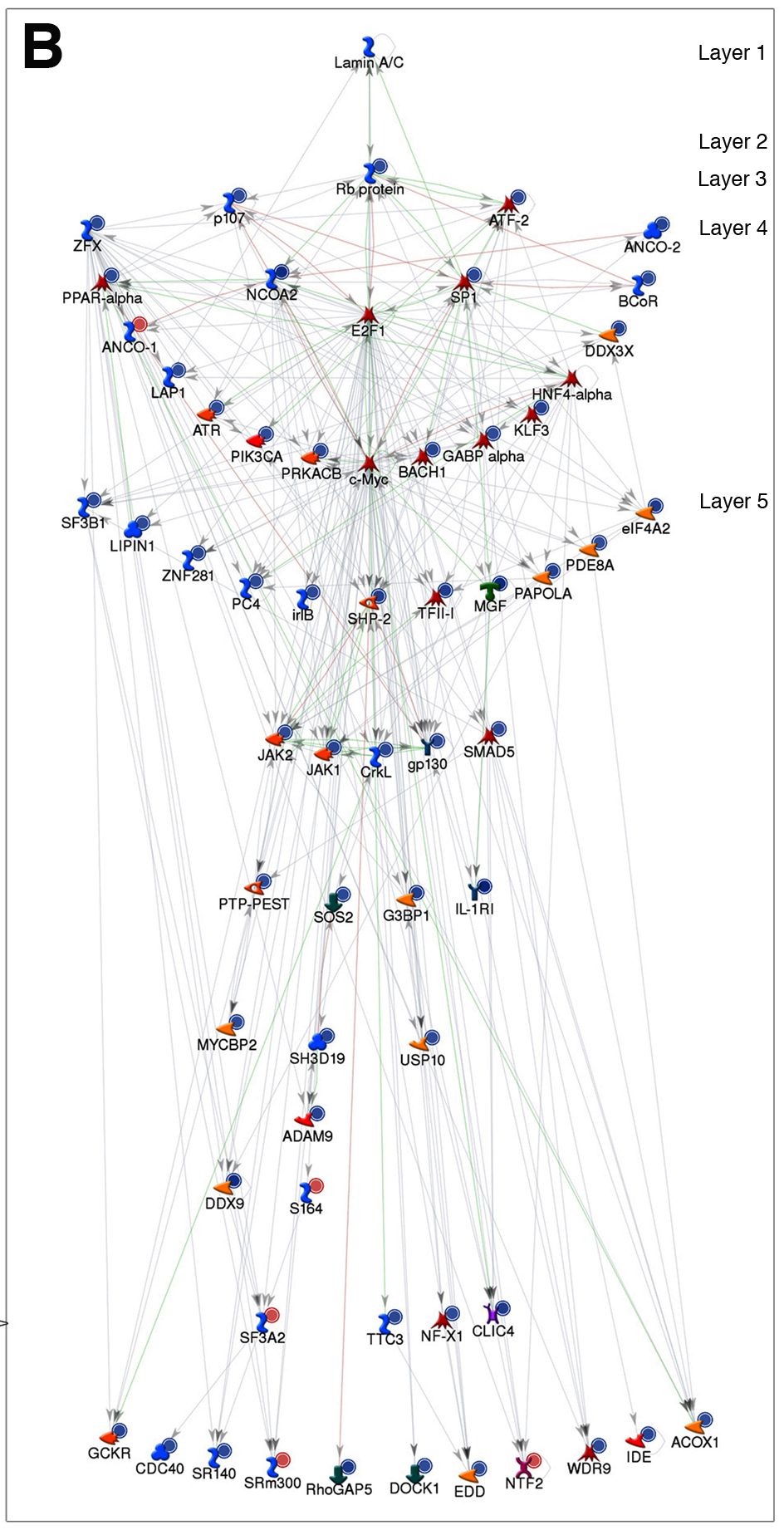

Supplement: Figure S2 — High magnification of Fig. 2, panel B. (3.89 MB TIF) [file pone.0011132.s002.tif]

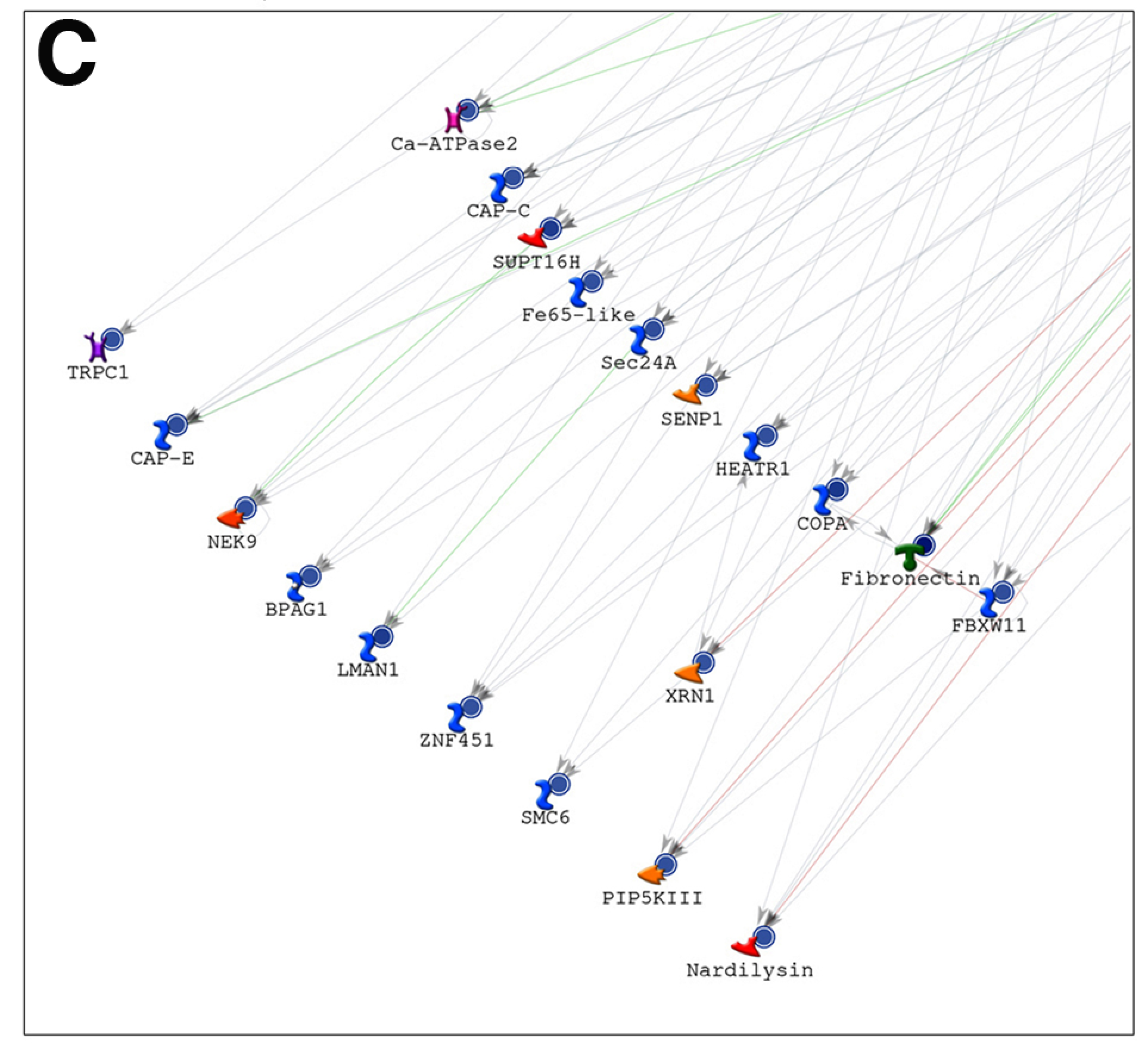

Supplement: Figure S3 — High magnification Fig. 2, panel C. (1.40 MB TIF) [file pone.0011132.s003.tif]

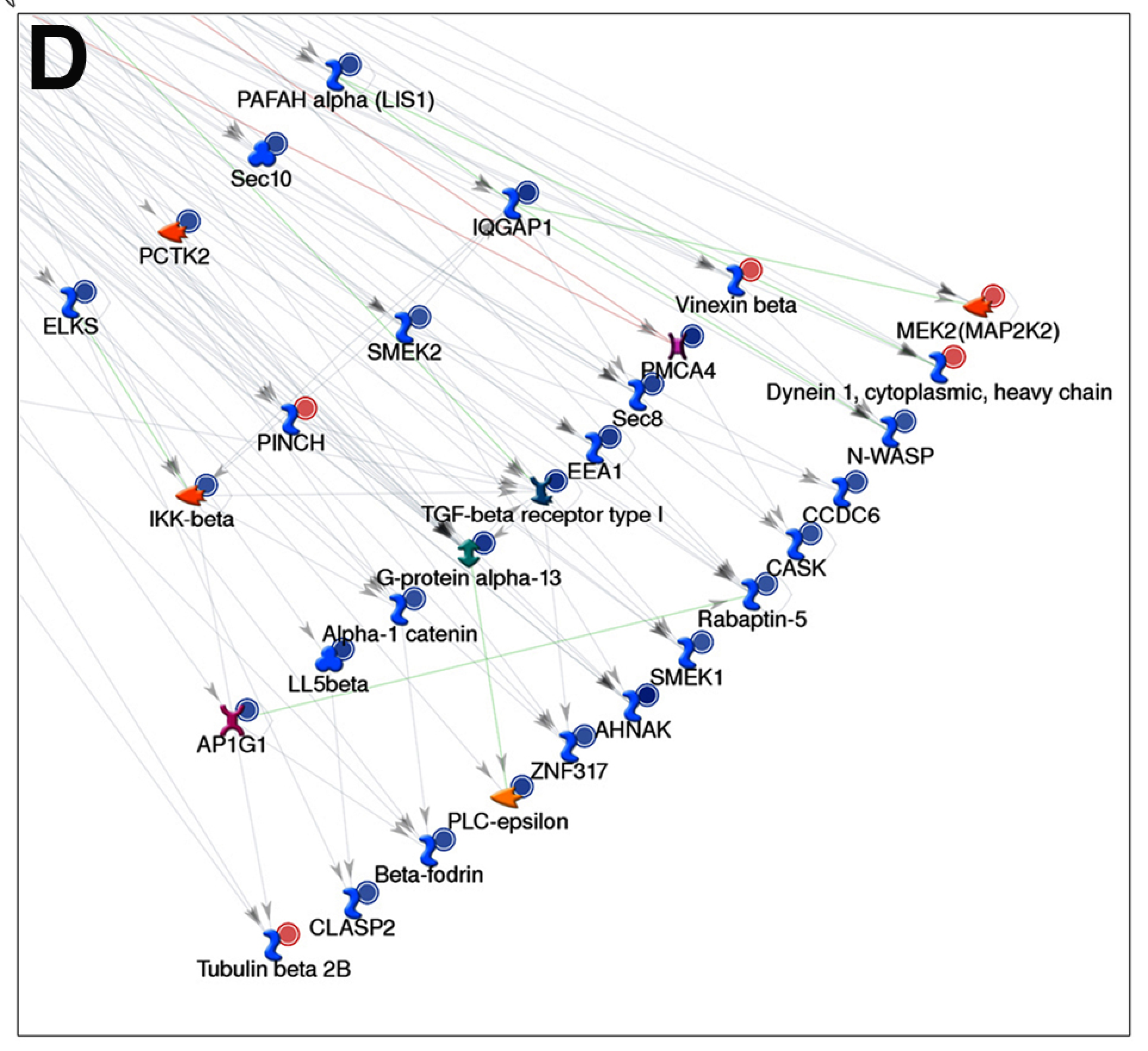

Supplement: Figure S4 — High magnification Fig. 2, panel D. (1.63 MB TIF) [file pone.0011132.s004.tif]

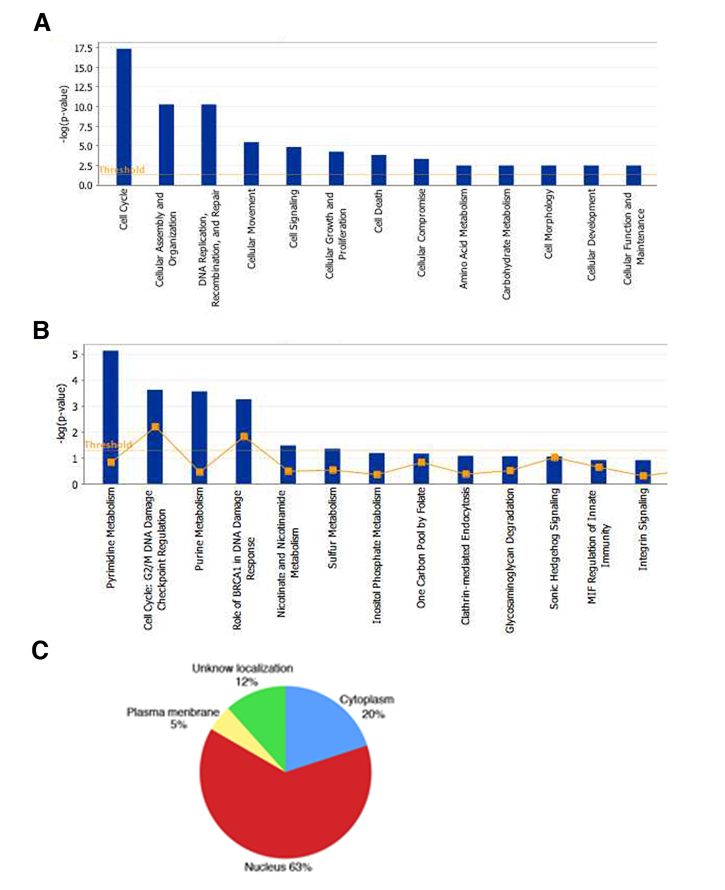

Supplement: Figure S5 — Genome-wide expression profiling of FTI-treated and untreated control fibroblast cultures. (A) Genes differentially expressed in normal fibroblasts treated with FTI compared to untreated normal fibroblasts were assigned to diverse cellular functions according to IPA, and (B) were associated with canonical pathways according to IPA. (C) Pie chart indicates the subcellular localization of the protein products of the differentially expressed genes according to information contained in the Ingenuity Knowledge Base. (0.37 MB TIF) [file pone.0011132.s005.tif]

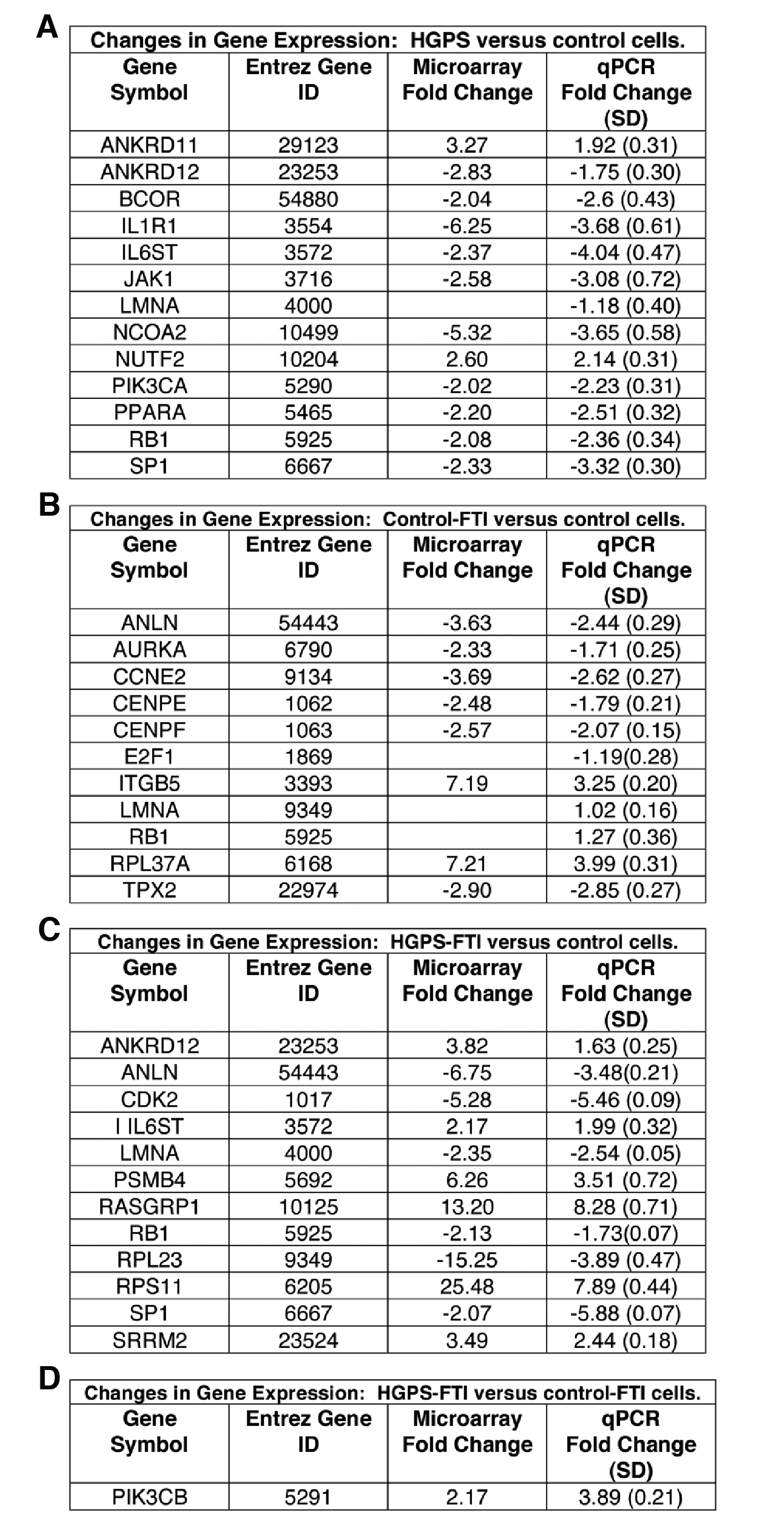

Supplement: Figure S6 — Validation of the microarray analysis by real-time RT-PCR. (A) Validation of a set of genes identified in microarray analysis comparing fibroblasts from subjects with HGPS to control. The mean value of expression for indicated genes measured using real time RT-PCR are indicated (SD: standard deviation; p<0.05), and microarray fold change (p<0.01) are shown. (B) Validation of a set of genes identified in microarray analysis comparing fibroblasts from control subjects with and without FTI treatment. (C) Validation of a set of genes identified in microarray analysis comparing fibroblasts from subjects with HGPS treated with FTI to untreated fibroblasts from control subjects. (D) Validation of the only gene differentially expressed between FTI-treated fibroblasts from subjects with HGPS to FTI-treated fibroblasts from control subjects. Fold changes measured by real-time RT-PCR and microarray analyses are indicated. (0.71 MB TIF) [file pone.0011132.s006.tif]

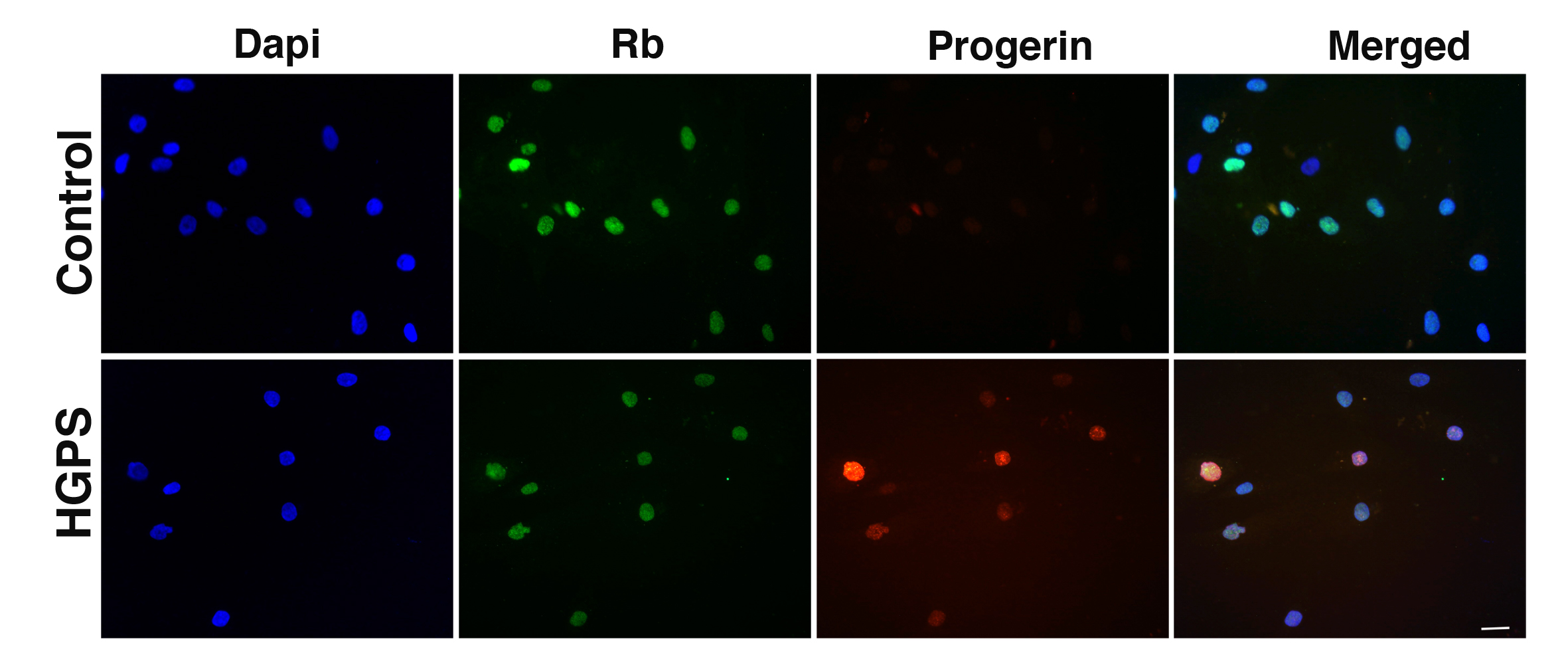

Supplement: Figure S7 — Distribution of progerin and Rb in cells from a subject with HGPS. Immunohistochemy was performed on fibroblasts from an unaffected control (GM03348) and a subject with HGPS (HGADFN003) at PPD 25 to 30. Cells were stained with anti-progerin antibody [31] (red) and anti-Rb monoclonal antibody (BD Biosciences Pharmingen) (green). Chromatin was stained with dapi. The triple merged signals are indicated. Scale bar, 20 µM. (1.31 MB TIF) [file pone.0011132.s007.tif]

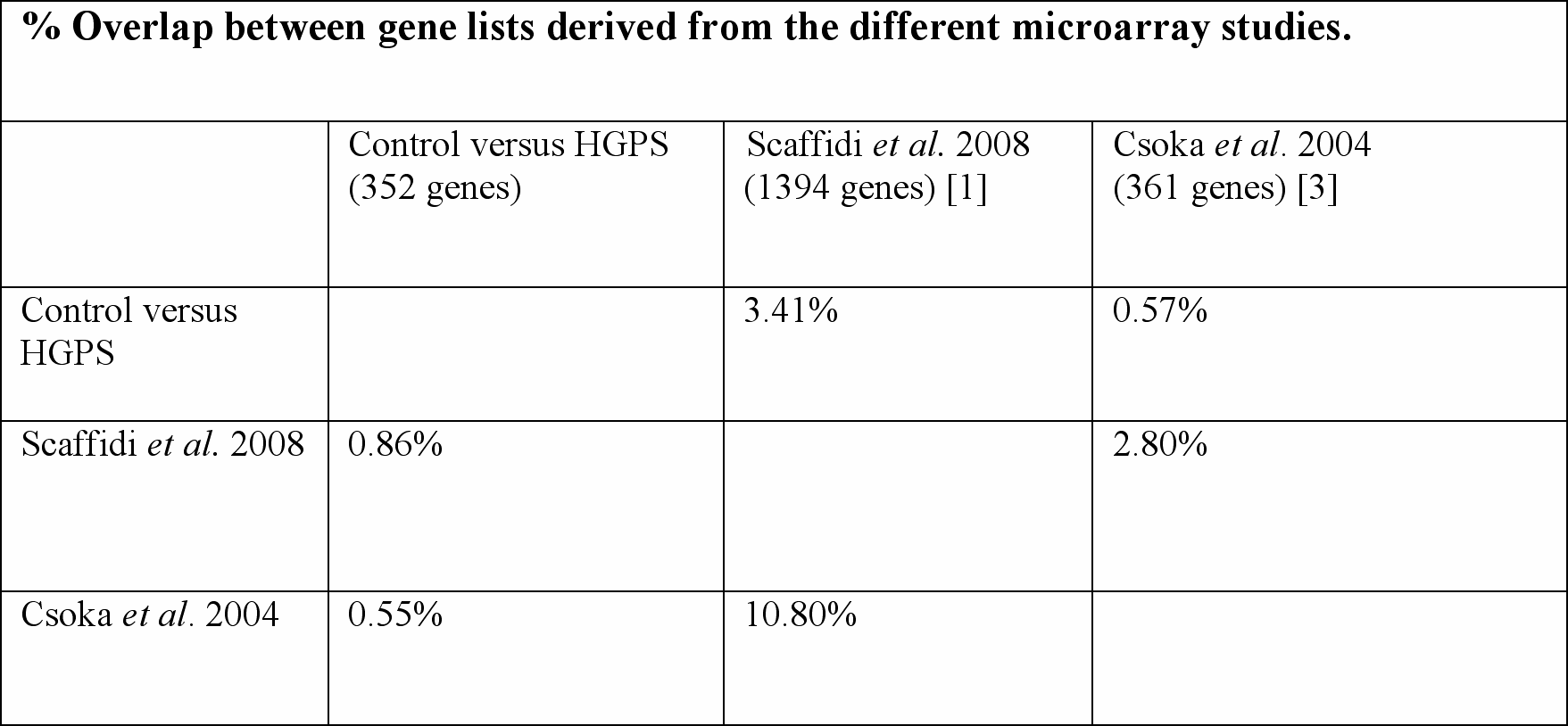

Supplement: Table S1 — Comparison of the differentially expressed genes in fibroblasts from subjects with HGPS from this study to the differentially expressed genes lists in studies by Scaffidi and Mitseli [28] and Csoka et al.[27] Control versus HGPS differentially expressed genes established after a statistical analysis using the t test with 1% significance and 2-fold cutoff were compared to the initial microarray analyses performed on three HGPS fibroblast strains (Coriell cell repositories) derived from patients at age 8 (AG11513), 9 (AG10750) and 14 years old (AG11498)[27]. This small overlap may be due to variation intrinsic to each cell, in addition to the fact that cells from subjects with HGPS exhibit increased variation in cellular phenotype with cellular age in vitro and with the donor's age. Our study was performed using five fibroblast cultures from five subjects with HGPS at age 2 to 4 years old, kindly provided by Progeria Research Foundation. Cells were collected at an early PPD (<25) when their growth rate remained similar to that of control fibroblast cultures. Phenotypic variations and different levels of progerin expression can contribute to the heterogeneity between fibroblasts from subjects with HGPS. We compared the control versus HGPS gene list with another study from Scaffidi and Mitseli that used a cellular model for HGPS by overexpressing progerin in normal immortalized fibroblasts [28] and found very little overlap. (0.07 MB TIF) [file pone.0011132.s008.tif]

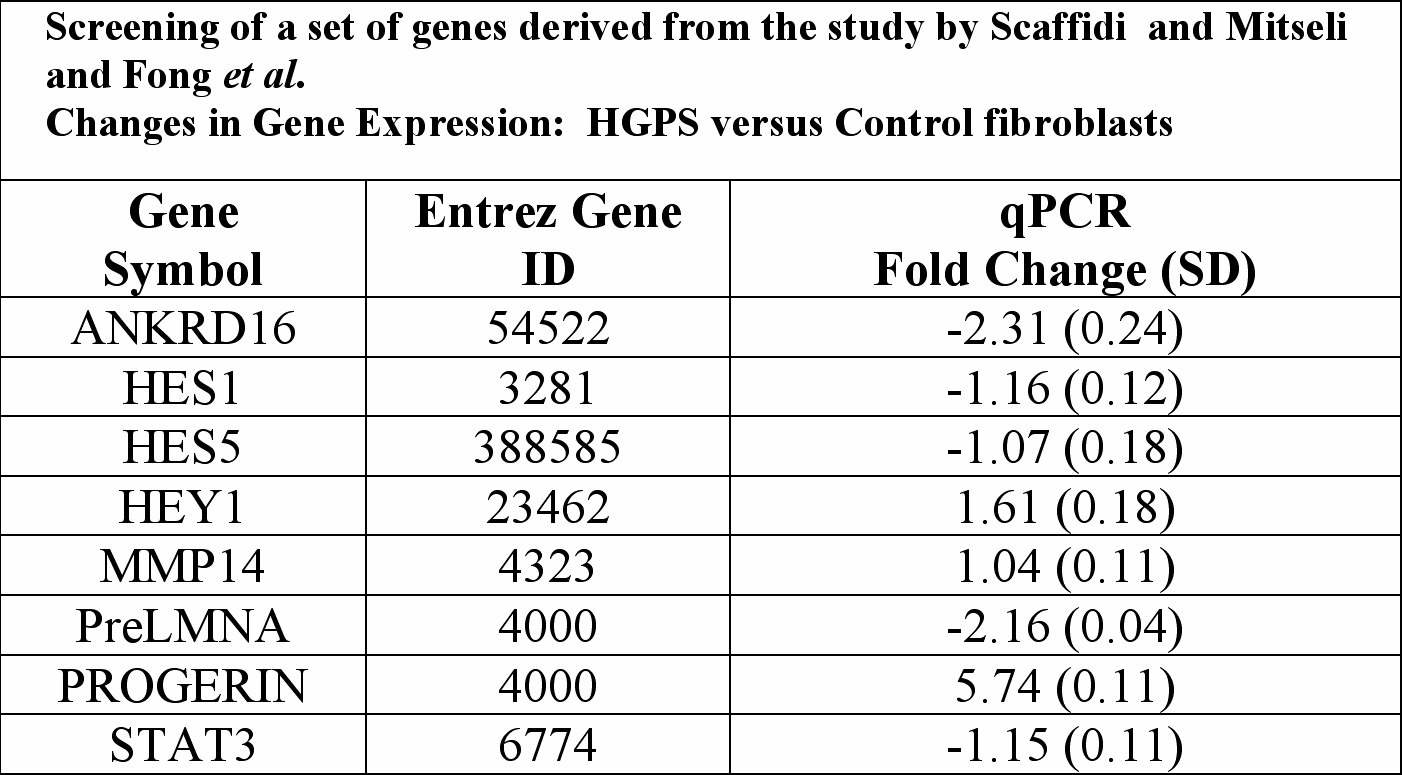

Supplement: Table S2 — Screening of a set of genes that were previously suggested to be perturbed in HGPS cells [28], [33]. Using the same olignucleotides for RT-PCR described by Fong et al 2009[2], we screened cultured fibroblasts from subjects with HGPS and from control individuals used in this study. The fold change between fibroblasts from subjects with HGPS and from control individuals in mRNA transcript for the corresponding genes are indicated with a P<0.05. (0.09 MB TIF) [file pone.0011132.s009.tif]

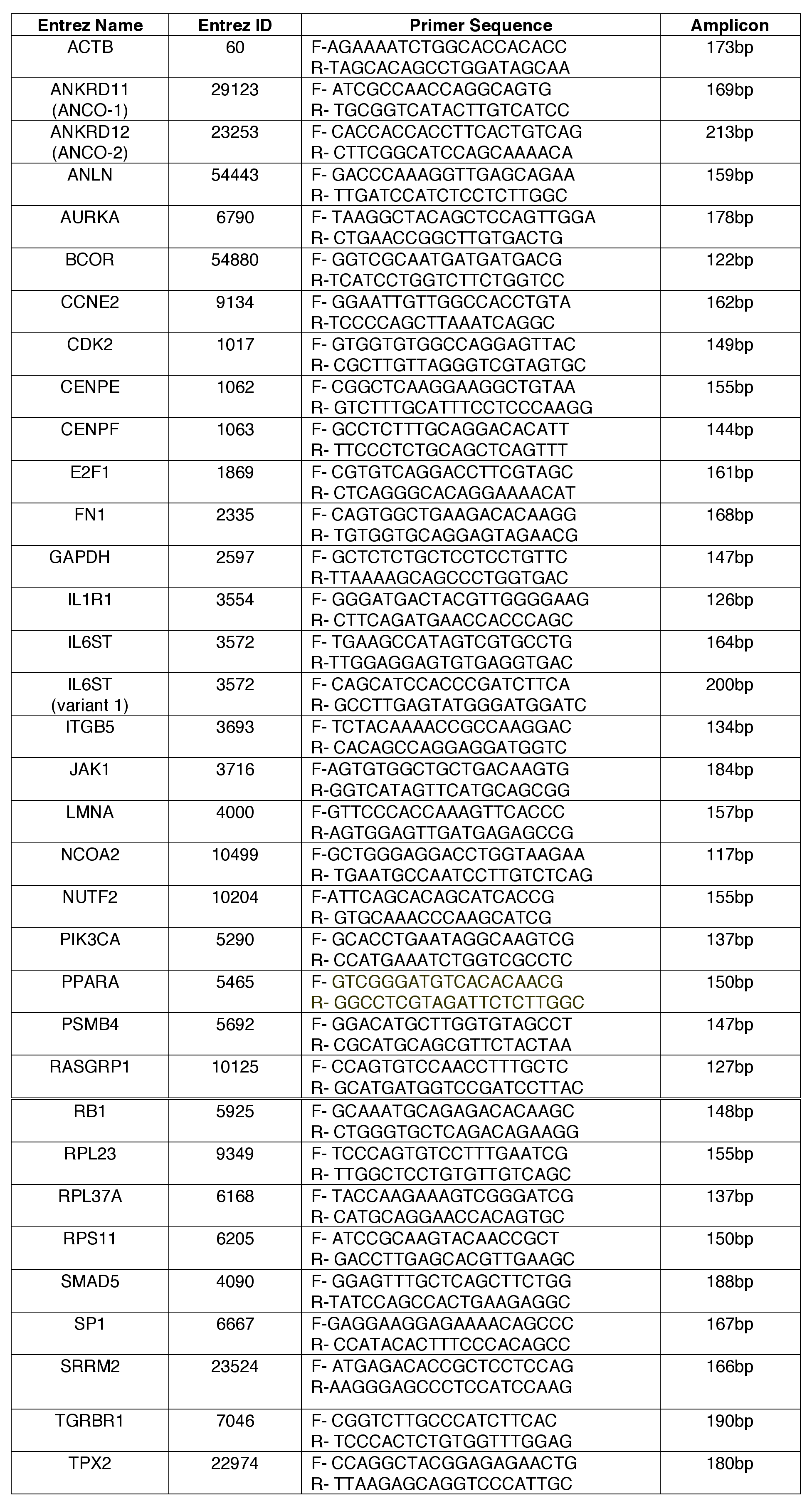

Supplement: Table S3 — List of primers used for real time PCR. (21.31 MB TIF) [file pone.0011132.s010.tif]
